# Advances in the Mechanism of Luteolin against Hepatocellular Carcinoma Based on Bioinformatics and Network Pharmacology

**DOI:** 10.7150/jca.80456

**Published:** 2023-04-09

**Authors:** Yunqi Han, Yunfeng Xiao, Lei Yu, Jing Chen, Xudong Yang, Hongwei Cui, Junqing Liang

**Affiliations:** 1 The Affiliated People's Hospital of Inner Mongolia Medical University/Inner Mongolia Autonomous Region Cancer Hospital, Hohhot 010050, China.; 2 Department of Pharmacy, Inner Mongolia Medical University, Hohhot 010110, China.; 3 Department of Pharmacy, Traditional Chinese Medicine Hospital of Inner Mongolia Autonomous Region, Hohhot 010020, China.; 4 Department of Medicine, Ordos Institute of Technology, Inner Mongolia Autonomous Region, Ordos 017000, China.; 5 Department of Urology, The Affiliated Hospital of Inner Mongolia Medical University, Hohhot 010050, China.

**Keywords:** Luteolin, Hepatocellular carcinoma, Flavonoids, Mechanism

## Abstract

As one of the most common malignant tumors, hepatocellular carcinoma (HCC) has a rising incidence rate and also seriously endangers human life and health. According to research reports, hepatitis B, hepatitis C, intake of aflatoxin in the diet, and the effects of alcohol and other chemicals can induce an increase in the incidence of liver cancer. However, in the current clinical treatment of HCC, most of the drugs are chemical drugs, which have relatively large side effects and are prone to drug resistance. Therefore, the development of natural compounds to treat HCC has become a new treatment strategy. Several studies have shown that flavonoids have shown outstanding effects and exhibit strong tumor growth inhibitory effects *in vivo* experimental studies. Luteolin, as a natural flavonoid, has anti-tumor, anti-inflammatory, anti-viral, anti-oxidation, immune regulation, and other pharmacological effects. The anti-cancer mechanism of luteolin mainly directly acts on tumor cells to inhibit their growth, induce cell apoptosis, reduce tumor tissue angiogenesis, regulate long non-coding RNA, affect immunogenic cell death, and regulate autophagy. As well as improving the curative effect of radiotherapy and chemotherapy and chemoprevention. In this study, we evaluated the function of luteolin in regulating cancer cell proliferation, migration, and invasion will summarize and analyze luteolin and its mechanism of regulating HCC to improve the role of luteolin in the clinical prevention and treatment of HCC.

## Introduction

Hepatocellular carcinoma is the deadliest and most common type of liver cancer, ranking sixth in the world in incidence and second in mortality 1,2. According to the GLOBOCAN 2020 database, it is estimated that there are 905,677 new liver cancer cases and about 830,180 death 3. This also shows that the mortality rate of liver cancer is gradually approaching the incidence rate. At this stage, the clinical early treatment of hepatocellular carcinoma (HCC) mainly consists of immunotherapy, surgical resection, radiofrequency ablation, and targeted therapy 4-7. Patients with early liver cancer can also be effectively treated by surgery, but many patients lose the best opportunity for surgical treatment because hepatocellular carcinoma is an invasive disease with a poor prognosis 8. It's worth noting that, flavonoids play an excellent anti-tumor effect, participate in and regulate the expression of a variety of tumor miRNAs, inhibit tumor cell mitosis, induce apoptosis, participate in immune responses, as well as inhibit the process of tumorigenesis through a variety of signal pathways 9-11. For example, luteolin, a derivative of flavonoids, is mainly found in fruits, vegetables, and natural plants and can be isolated from a variety of traditional Chinese medicines 12. Luteolin also can regulate miRNA expression in different cancer to affect the cancer progression. At present, LUT has a good anti-cancer application prospect, and its derivatives have potential anti-cancer effects, and Ma Jun, Yoo Ho Soo, and others have proved that luteolin can affect the survival of a variety of cancer cells such as gastric cancer and colon cancer 13,14. Nevertheless, more studies are needed to provide a better understanding of the mechanism of cancer treatment using luteolin. For this reason, based on the structure and pharmacological effects of luteolin, this article explored and reviewed the mechanism of luteolin's anti-growth, proliferation, apoptosis, anti-oxidative stress, angiogenesis, and related molecular signaling pathways of hepatocellular carcinoma, to provide new strategies for the treatment of hepatocellular carcinoma.

## The pharmacological activity of luteolin

### The structure of flavonoids

Flavonoids are a class of planting metabolites with pharmacological activity. It is a compound with 2-phenyl chromone as the core. It generally refers to a series of compounds formed by connecting two benzene rings (A and B rings) with phenolic hydroxyl groups through the central 3 carbon. The basic skeleton is C6-C3-C6, mainly found in the natural plant kingdom 15. According to the characteristics of the core structure, the structure of flavonoids can be modified by different substituents to obtain discrete derivatives, resulting in different properties 7,16. The main structural types of its derivatives are: including flavones, flavonols, isoflavones, flavanones, flavanonols, dihydro isoflavones Dihydro-isoflavones, flavan-3-ols, flavan-3,4-diol, anthocyanidins, chalcones (chalcones) and dihydrochalcones (dihydrochalcones), etc.** (Table 1)**.

**Figure 1:**
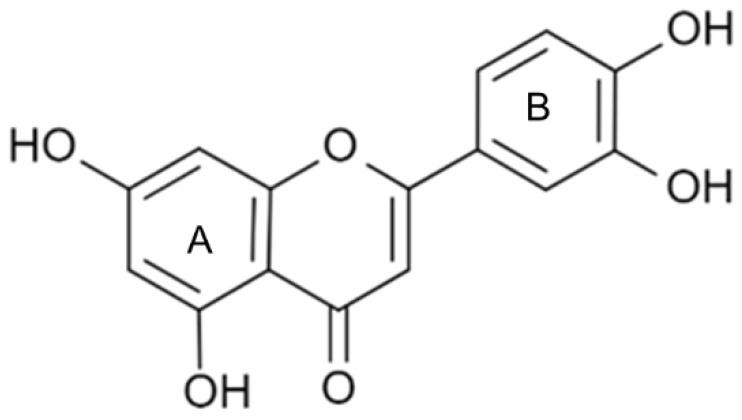
The chemical structure of luteolin. It is a compound with 2-phenyl chromone as the core. It refers to a series of compounds formed by connecting two benzene rings (A and B rings) with phenolic hydroxyl groups through the central 3 carbon. The basic skeleton is C6-C3 -C6.

The chemical structure of luteolin (3',4',5,7-tetrahydroxyflavonoids, Luteolin, LUT) **(Figure 1)**, which is a representative derivative of dihydroflavonoids, mainly found in plants such as Verbenaceae, Apiaceae and other natural medicinal materials and vegetables 17. In recent years, research on the structure of Luteolin has shown that the structure of LUT contains two aromatic rings (ring A and ring B) connected through three carbon atoms in the center 18. Therefore, the structural characteristics of LUT also determine the nature of its action. Several pharmacological studies have proved that LUT has a wide range of biological activities, including anti-inflammatory, anti-allergic, anti-cancer, antioxidant, or pro-oxidant. In recent years, flavonoids have played a major role in tumor treatment, and luteolin as a flavonoid derivative has also received research attention 19. Just as Kapoor S 20 and Lee EJ 21 proposed that luteolin can inhibit EGF-mediated MAPP, ERK, and AKT signal pathways by reducing the expression of epidermal growth factor (EGF) mRNA.

### The pharmacological effects of luteolin

#### The effect of LUT on scavenging free radicals and anti-oxidation

**Figure 2:**
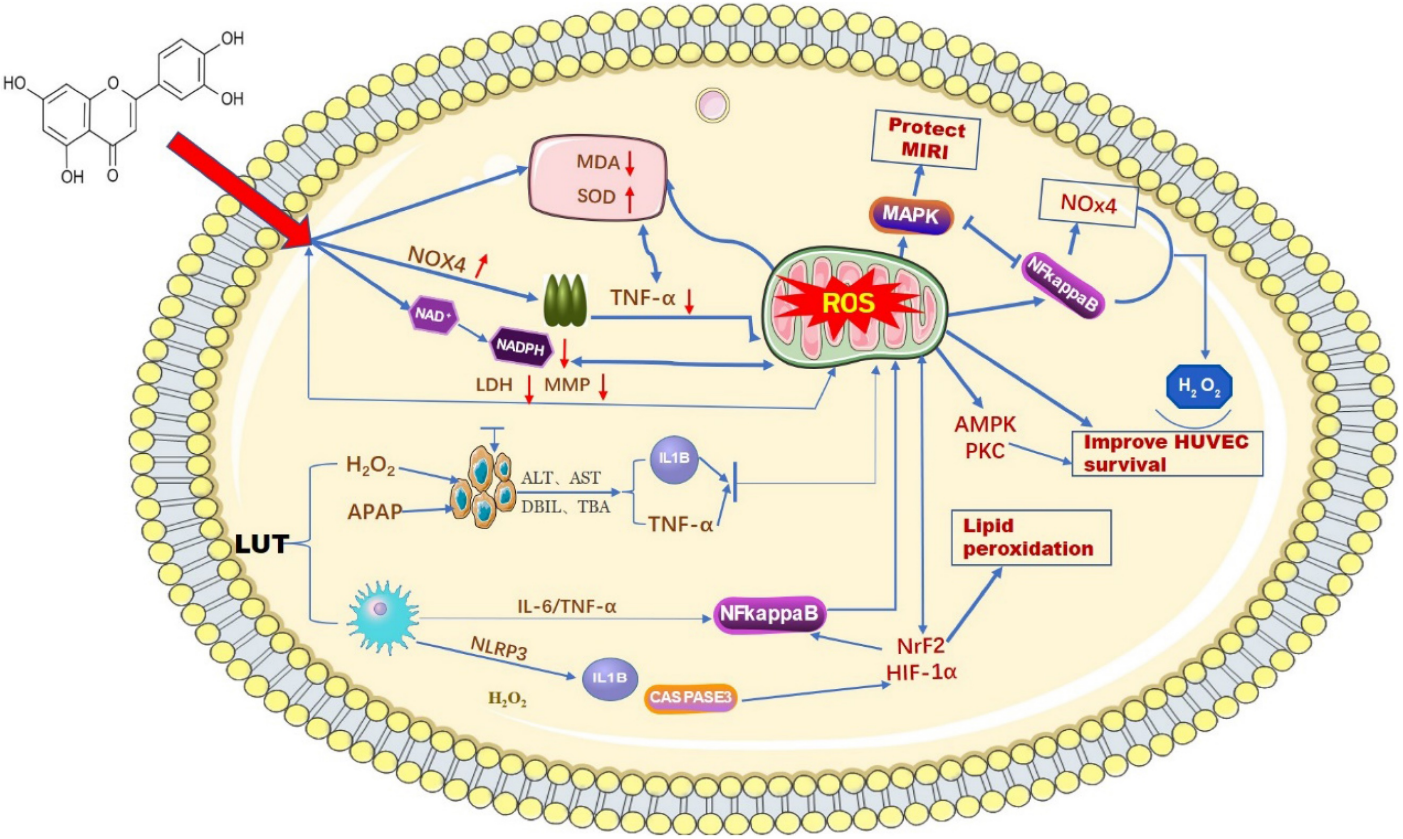
Schematic diagram of the influence of LUT on oxygen free radicals. Luteolin has a wide range of anti-cancer effects. Among them, it can affect the content of ROS in mitochondria by reducing various biochemical indicators and signaling factors in liver cancer cells.

ROS, as important molecules that induce oxidative stress in the body, can be generated in hepatocytes and macrophages when cells undergo an inflammatory response 22. The imbalance of stromal cells, the immune microenvironment, and some other biological pathways are also closely associated with ROS 23. Luteolin, as a natural flavonoid compound, contains a certain number of phenolic hydroxyl groups in its structure, which provides it with strong reducibility and antioxidant properties. Luteolin exerts an antioxidant effect on biological systems by directly inhibiting the formation of reactive oxygen species, activating antioxidant enzymes, and promoting antioxidant defenses 24,25. Balanchine 26 et al. demonstrated that luteolin can stabilize the leakage of intracellular antioxidant defense systems GSH, CAT, and SOD and reduce the activity of MDA, thus reducing the production of ROS and ultimately protecting mitochondria from damage in cardiac myocytes. According to reports. H2O2 can induce oxidative stress in human umbilical vein endothelial cells (HUVECs), and the production of ROS superoxide can be reduced after an intervention by luteolin 27. At the same time, luteolin protects HUVECs from TNF-α-induced oxidative stress and inflammation through its effects on Nox4**/**ROS-NF-κB and MAPK pathways 28. Research data show that luteolin has been found to reduce the generation of ROS by reducing the leakage of LDH and the reduction of mitochondrial membrane potential, thereby alleviating OTA-induced oxidative stress and lipid peroxidation through NrF2/HIF-1α 29. Prateheshkumar 30. Showed that luteolin inhibits ROS production, NADPH oxidase (NOX) activation, glutathione consumption, and lipid peroxidation in a dose-dependent manner **(Figure 2)**.

#### Influence of LUT on inflammation

**Figure 3:**
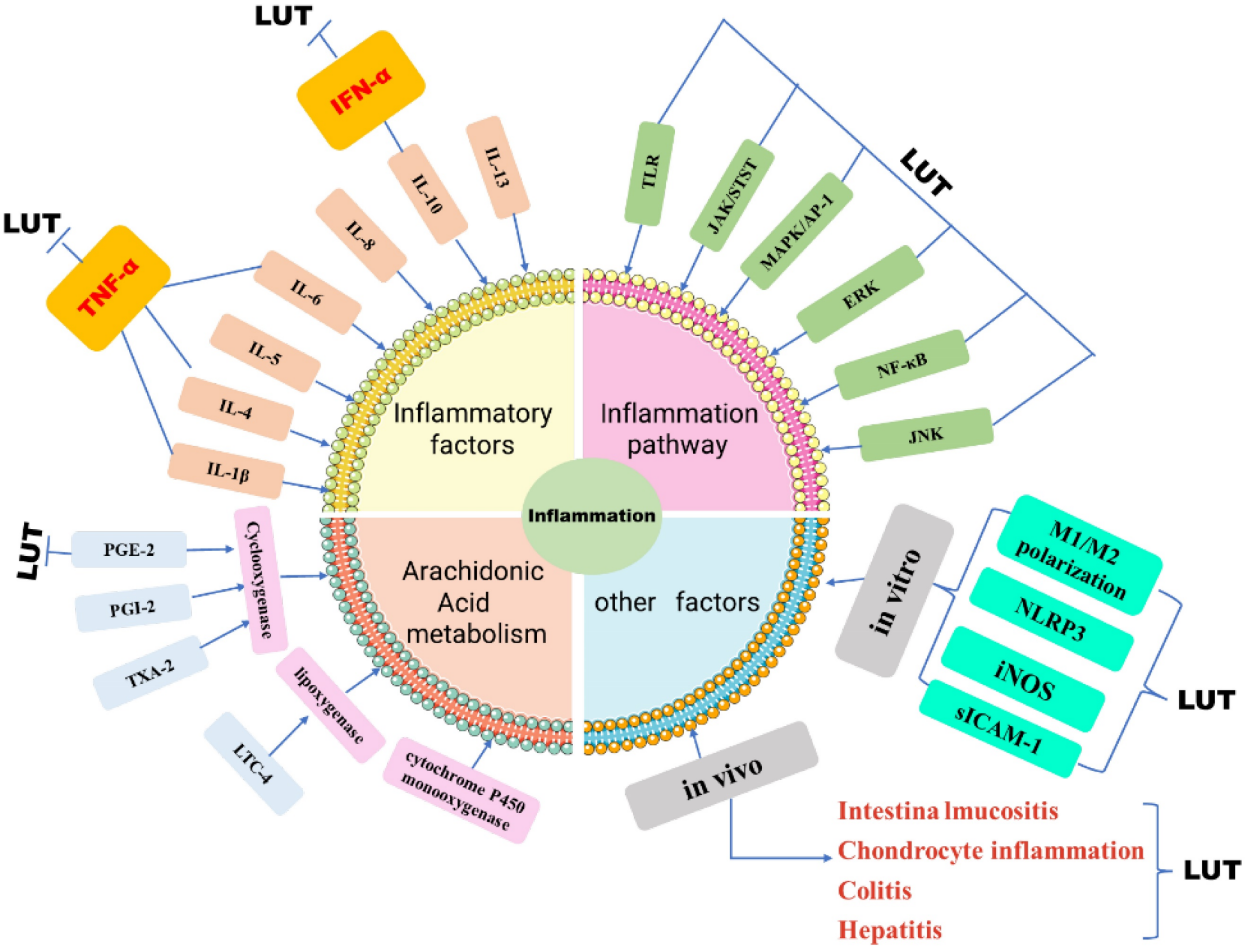
Schematic diagram of LUT regulating inflammation. The role of inflammatory response in tumors cannot be underestimated. Luteolin reduces the expression of inflammatory factors in tumor cells by influencing inflammatory factors, inflammatory signal pathways, and arachidonic acid, *in vivo* and *in vivo*.

Part of the anti-inflammatory effect of luteolin is achieved by regulating inflammatory mediators, and it has been demonstrated to regulate inflammatory factors and inflammatory mediators in a variety of *in vivo* and *in vitro* models 31** (Figure 3)**. Luteolin can inhibit IL-1β, IL-4, IL-5, IL-6, IL-8, IL-10, IL-13, TNF-α, interferon (IFN)-β and granulocyte-macrophages Cell colony-stimulating factor (GM-CSF), and can increase the level of anti-inflammatory cytokine IL-10 32. In addition, luteolin can also inhibit chemokines that can control the migration and localization of immune cells such as CCL2, CXCL2, CXCL8, and CXCL9 33. And by regulating diverse signaling pathways, such as nuclear transcription factor NF-κB, MAPK/AP-1, JAK-STAT, and TLR signaling pathways, etc 34. At the same time, Cho Young 35. Concluded that luteolin can reduce the production of NO by reducing the synthesis of nitric oxide synthase (iNOS). In macrophages induced by lipopolysaccharide plus interferon-γ stimulation and interleukin 4 (IL-4) stimulation, luteolin changed the M1/M2 polarization of macrophages and was down-regulated by p-STAT3 the up-regulation of p-STAT6 exerts anti-inflammatory effects 36. Studies have also shown that in the bone marrow-derived macrophages of SD rats, Luteolin inhibits the production of TNF-α and IL-6 in a dose-dependent manner, and shortens the half-life of TNF-α and IL-6mRNA 37. In addition, luteolin can also inhibit inflammation by changing the activities of histone decarboxylase (HDAC) and acetylene (HAT) 38. Luteolin-mediated Coxsackie virus B3 (CVB3) can be achieved. Luteolin inhibits the phosphorylation of p38 MAPK, JNK, and ERK in CVB3, thereby inhibiting NF-κB nuclear translocation and subsequently reducing inflammation in CVB3-infected cells and the expression of cytokines 39. In IL-1β-induced rat chondrocyte inflammation, Luteolin inhibits the phosphorylation of NF-kappaB *in vivo* and attenuates the pathogenesis of osteoarthritis model rats 40. In the rat model of acute pneumonia, luteolin treatment reduced the dry-to-wet weight ratio of lung tissue and decreased the total number of serum white blood cells in a dose-dependent manner. These studies proved that luteolin partially inhibited the neutrophil ring Adenosine phosphate (cAMP-PDEs) or PDE4 activity and the expression of vascular cell adhesion molecule (VCAM-1) and intracellular cell adhesion molecule (sICAM-1) in microvascular endothelial cells to inhibit inflammation 41. Arachidonic acid acts as the direct precursor of prostaglandin (PGI-2), thromboxane A2 (TXA-2), and leukopenia (LTC-4) 42. In the inflammation reaction, PGE2 can play a pro-inflammatory effect by regulating the differentiation of immune cells or increasing the expression of related cytokines, thereby exacerbating the inflammatory response 43. However, studies have confirmed that luteolin has varying degrees of influence on the two pathways of cyclooxygenase (COX) and lipoxygenase (LOX), among which the mechanism of action is to regulate PGE-2, IFN-α, and β 44.

#### The impact of LUT on tumor

Luteolin is present as a flavonoid in vegetables, plants, and fruit. Take part in the fight against various human malignant tumors. In the mechanism studies published so far, most of the effects of luteolin in alleviating breast cancer, colon cancer, pancreatic cancer, lung cancer, kidney cancer, gastric cancer, and other tumors are by inhibiting the proliferation of tumor cells. Reduce the stimulation of carcinogens, activate cell cycle arrest, etc. to play a role. In addition, luteolin is also involved in the regulation of genes and proteins to induce tumor cells to induce apoptosis through distinct signal pathways and block the development of cancer *in vivo* and *in vivo*** (Table 2)**.

## The effect of LUT on HCC

Luteolin's anti-hepatocellular carcinoma activity is linked to its influence on various signal transduction pathways and cytokines in liver cancer cells. Several experimental studies have proved that luteolin can prevent the spread, metastasis, and cell cycle arrest of hepatocellular carcinoma, promote cell differentiation and angiogenesis, and also promote the apoptosis of malignant cells. A large number of *in vivo* and *in vivo* evaluations show that luteolin promotes tumor cell apoptosis, inhibits tumor cell growth cycle, tumor cell migration and invasion, etc., and its anti-tumor development potential is huge.

### The effect of LUT on the proliferation of HCC cells

**Figure 4:**
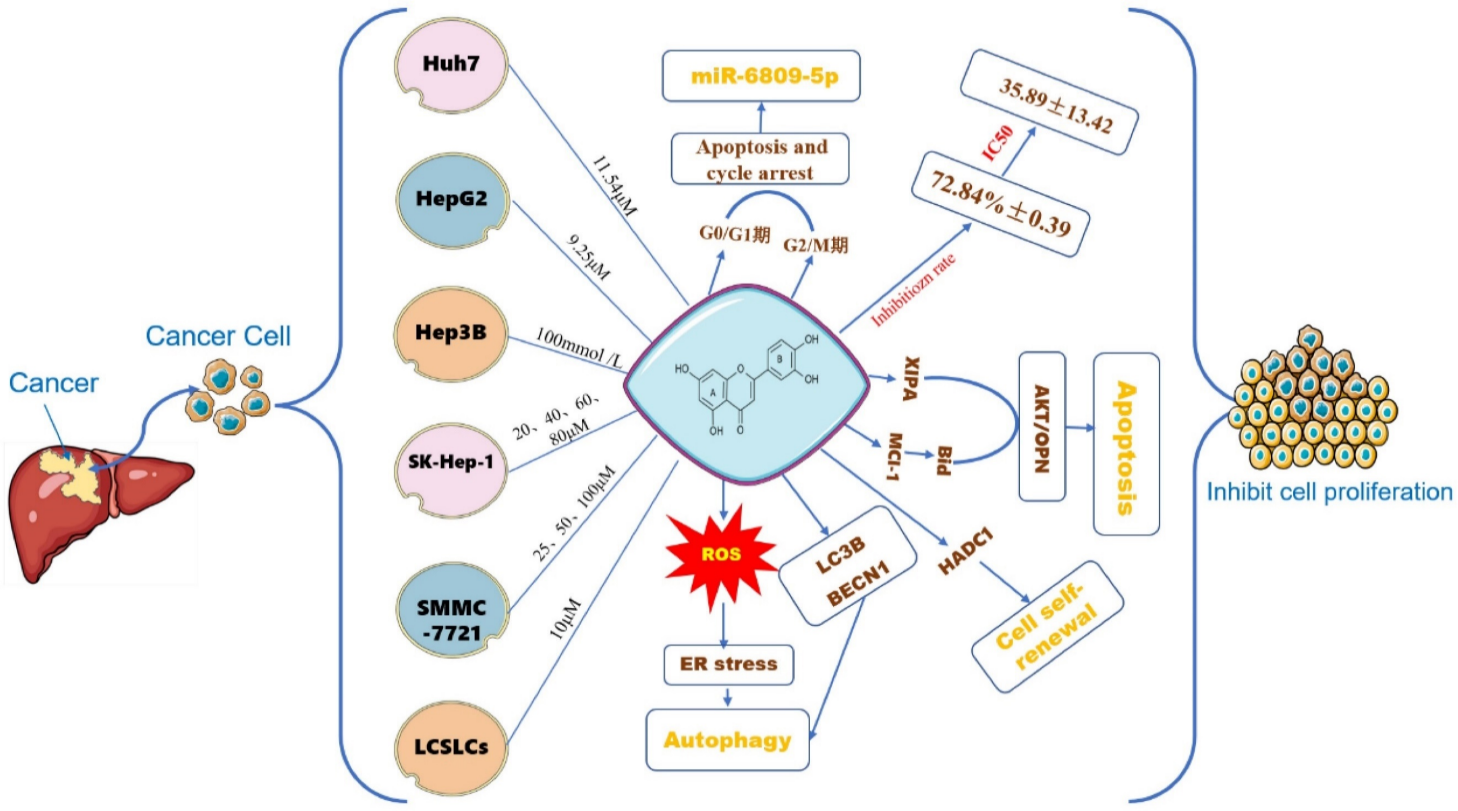
The effect of LUT on the growth of different HCC cells. Note: The figure above illustrates the effect of different concentrations of luteolin on inhibiting the proliferation of different types of liver cancer cells such as Huh7, HepG2, Hep3B, SK-Hep-1, PC-3, LCSLCs, etc. Luteolin 11.54μM can promote cell apoptosis by influencing the polarization of Huh7 cells in the G0/G1, G2/M phase and regulating the expression of miR-6809-5P gene; oxidative stress is another important part of tumor development. On the one hand, it directly or indirectly affects the proliferation of tumor cells. Luteolin has the effect of affecting ROS, and finally induces ER stress and oxidative stress; the self-renewal of cancer cells plays a key role in the occurrence and metastasis of tumor cells. Luteolin can regulate the self-renewal of LCSLCs.

Cell proliferation is one of the important physiological functions of cells, and an essential life characteristic of organism growth, development, reproduction, and heredity 55. Similarly, the proliferation of cancer cells is another important step in tumorigenesis and development. The antitumor activity of luteolin has been investigated in various cancer cells. Luteolin can induce cell cycle arrest and cell apoptosis, and inhibit proliferation and metastatic progression. Based on the rapid progress of flavonoids, their effects on tumor cell proliferation have also been extensively studied **(Figure 4)**. Yang P W 56 et al, treated HuH7 and HepG2 hepatoma cells with different concentrations of luteolin for 24-120h, the results showed that luteolin inhibited the proliferation of hepatoma cells in a dose-dependent manner, and the result of HepG2 was more obvious than that of HuH7. Luteolin 9.25 ± 1.67 μM can achieve half the inhibitory effect, while in Huh7 cells 11.54 ± 2.32 μM can achieve half of the inhibitory effect. Experimental studies by Chang J 57 and others suggest that luteolin has an inhibitory rate of 72.84% ± 0.39 on human liver cancer cells Hep3B at a concentration of 100mmol/L, which has a significant inhibitory effect on cell proliferation. Hepatocyte growth factor (HGF), also known as scattering factor (SF) and its receptor c-Met tyrosine kinase is in charge of the proliferation of a variety of cancer cells. Researchers have found that luteolin and other flavonoids can regulate HGF factors it reduces the survival rate of HepG2 cells 58. 100μmol/L luteolin inhibits the proliferation of liver cancer cells by down-regulating the miRNA expression of the proliferation-related genes LETM1, URG11, PICK1, and CyclinD1 in HepG2 liver cancer cells, suggesting that luteolin may inhibit cell proliferation 59. Im Eunji 60 et al, used 20, 40, 60, and 80 µM luteolin to interfere with human hepatocellular carcinoma SK-Hep-1 cells and mouse normal hepatocyte AML12 cells, and the results showed that luteolin significantly reduced SK-The viability of Hep-1 cells is dose-dependent.

To detect the effect of luteolin on SMMC-7721 cell autophagy, the formation of autophagosomes was observed using transmission electron microscopy, the number of intracellular autophagosomes after treatment with 25,50, or 100 µM luteolin for 48 h increased compared with cells treated with 0 µM luteolin 61. Furthermore, luteolin increased the number of autophagosomes within cells, promoted the conversion of LC3B-I to LC3B-II, and increased the expression of Beclin 1, a phenomenon that, finally, was altered by the addition of an autophagosome inhibitor. 10μM luteolin can inhibit the activity and expression of histone deacetylase-1 (HADC1) in liver cancer stem-like cells (LCSLCs), thereby influencing the self-renewal of LCSLCs 62. 5- 10 µmol/L luteolin induces oxidative stress and ER stress in p53-null Hep3B cells, and only induces autophagy in Hep3B cells, enhancing cell viability 63.

### The effect of LUT on HCC cell apoptosis

Cell cycle regulation requires the cooperation of a large number of intracellular and extracellular signals, without proper signals, cells will be unable to move from one stage to the next, this phenomenon is called cell cycle arrest 64. Cell cycle arrest helps to maintain the stability of genes, and gene mutations that regulate the cell cycle plays a major role in tumorigenesis. When the cell cycle is normal, if DNA damage occurs, the cell cycle stops at the corresponding checkpoint, and the cell cycle block provides extra time for the cell to repair the damage, thereby reducing the occurrence of mutations and avoiding the occurrence of tumors 65, studies have demonstrated that treatment with luteolin and apoptosis-inducing ligand (TRAIL) has a synergistic effect and mechanism on Huh7 cells 66. This is consistent with the study of Wu B 67 that luteolin induces autophagic flux of human liver cancer cells, significantly inhibits the expression of death receptor 5 (DR5) in the process of tumor apoptosis, and effectively enhances Apoptosis induced by TRAIL. CyclinD1 protein is a key protein for cells to transform from the G1 phase to the S phase. Studies have shown that luteolin regulates cell cycle arrest by down-regulating the expression of CyclinD1 gene mRNA in liver cancer cells 68, this is in line with the discovery of Shi Dongdong et al. that luteolin-blocked MCF-7 cells in S phase 69. Luteolin can promote the apoptosis of hepatoma cells by up-regulating the expression of p-JNK protein in HepG2 cells, and it can also induce mitochondrial autophagy in HepG2 cells by down-regulating the expression of Bcl-2 56. Studies have shown that treatment with 40 μmol/L luteolin for 2 hours can promote the activity of caspase-3 and -8 by degrading the x-linked apoptosis protein inhibitor (XIAP) and inhibiting the activity of protein kinase C (PKC) 70. Pretreatment of SMMC-7721 and Bel-7402 liver cancer cells with 50μM luteolin showed that the level of apoptosis factor Bax was up-regulated, the anti-apoptotic factor Bcl-2 was down-regulated, caspase-3 enzyme was activated, and mitochondrial membrane potential was reduced and induced Liver cancer cells to undergo apoptosis and exert their anti-liver cancer function 71. In addition, the combination of metformin and luteolin sympathetically protects liver toxicity induced by carbon tetrachloride, and its effect may be related to the anti-apoptotic pathway Nrf2/HO-1 72.

### The effect of LUT on HCC angiogenesis

**Figure 5:**
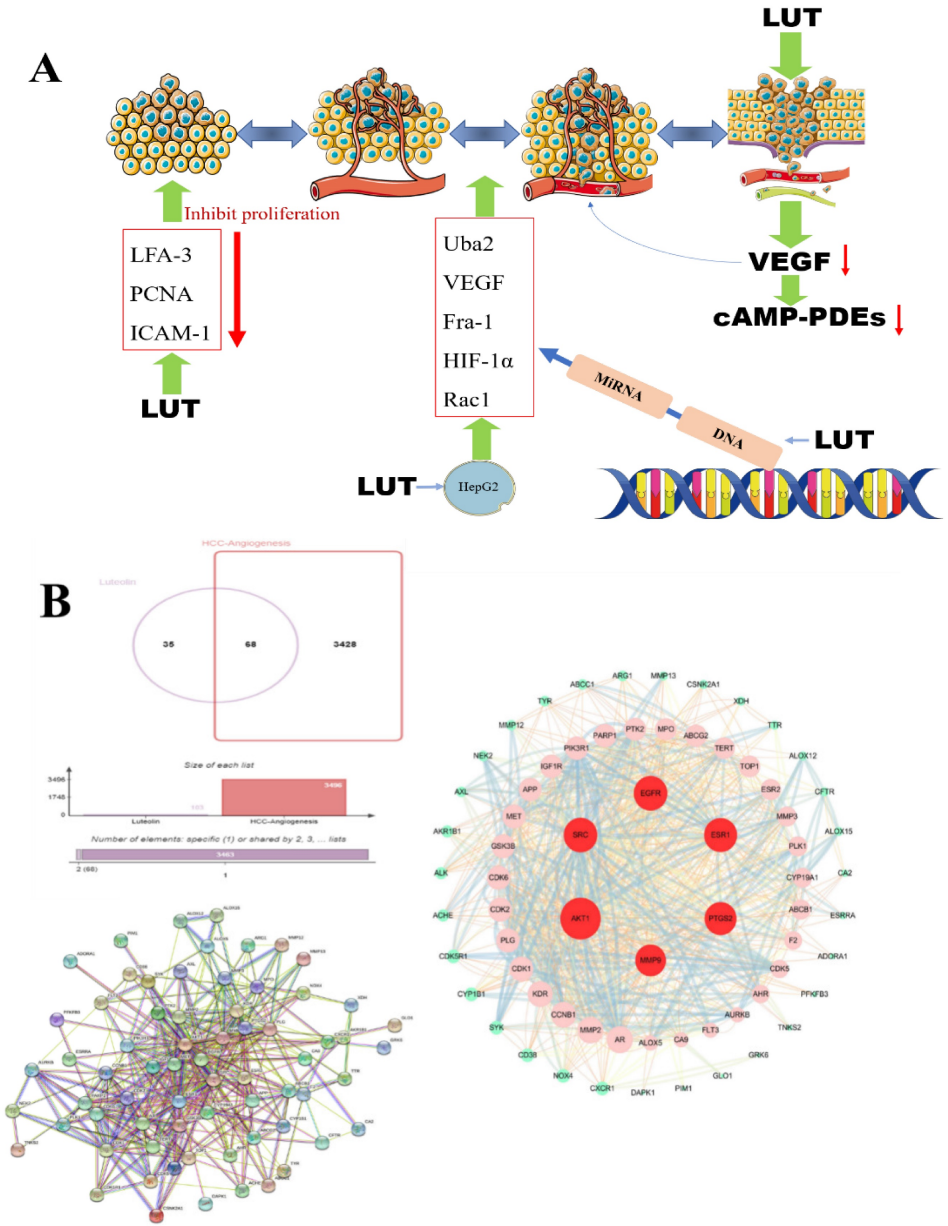
** A.** The effect of LUT on the angiogenesis of liver cancer. The production of VEGF (vascular endothelial growth factor, vascular endothelial growth factor) is reduced under the action of luteolin and further down-regulates the cyclic adenosine monophosphate (cAMP-PDEs) of neutrophils to inhibit tumor angiogenesis; **B.** LUT regulates tumor cells A variety of gene expressions and DNA inhibit tumor angiogenesis; research on the potential targets of luteolin for hepatocellular carcinoma angiogenesis, PPI shows that luteolin can inhibit angiogenesis through targeted regulation of AKT1, SRC, EGFR, ESR1, etc.;

Angiogenesis, the process of forming new blood vessels and blood supply structures, is one of the important mechanisms of tumor growth and metastasis 73. Vascular endothelial growth factor (VEGF), platelet-derived growth factor (PDGF), angiotensin-converting enzyme (Ang) and fibroblast growth factor (FGF) are key factors in angiogenesis. High levels of circulating vascular endothelial growth factor in patients with hepatocellular carcinoma are closely associated with tumor angiogenesis 74. Therefore, preventing/blocking the rapid formation of blood vessels is an effective way to prevent hepatocellular carcinoma. Rat microvascular endothelial cells have been shown to express high levels of cAMP-PDEs, especially PDE4, and further studies have shown that lignocaine has a dose-dependent inhibitory effect on the activity of endothelial cAMP-PDEs or PDE4 41. Luteolin can regulate the mRNA expression of pro-proliferative genes, pro-apoptotic genes, and angiogenic molecules Uba2, VEGF, Fra-1, HIF-1α, and Rac1 in hepatocellular carcinoma HepG2 cells, thereby inhibiting the proliferative activity and angiogenesis of hepatocellular carcinoma cells 75. Studies have reported that luteolin downregulates lymphocyte function-related molecules (LFA-3) and PCNA and upregulates intercellular adhesion molecule-1 (ICAM-1) in a way that inhibits tumor angiogenesis and tumor cell proliferation to achieve the anti-tumor effect of LUT 76 **(Figure 5A)**. SRC and EGFR can be considered the main genes to prevent angiogenesis and inhibit the growth of tumor cells. Zhulin Wu 77 et al. studied 207 patients with hepatocellular carcinoma and found that luteolin and quercetin could play a therapeutic role through MAPK, JAK-STAT, and other pathways, and the key targets included SRC, EGFR, VEGFA, PIK3R1 and so on. Based on the above research results, we verified by network pharmacology technology the potential targets of luteolin in the treatment of hepatocellular carcinoma angiogenesis include AKT1, SRC, EGFR, ESR1, MMP9, and PTGSR** (Figure 5B)**.

## Mechanism and clinical application of luteolin in the treatment of HCC

### Bioinformatics study of luteolin in the treatment of HCC

**Figure 6:**
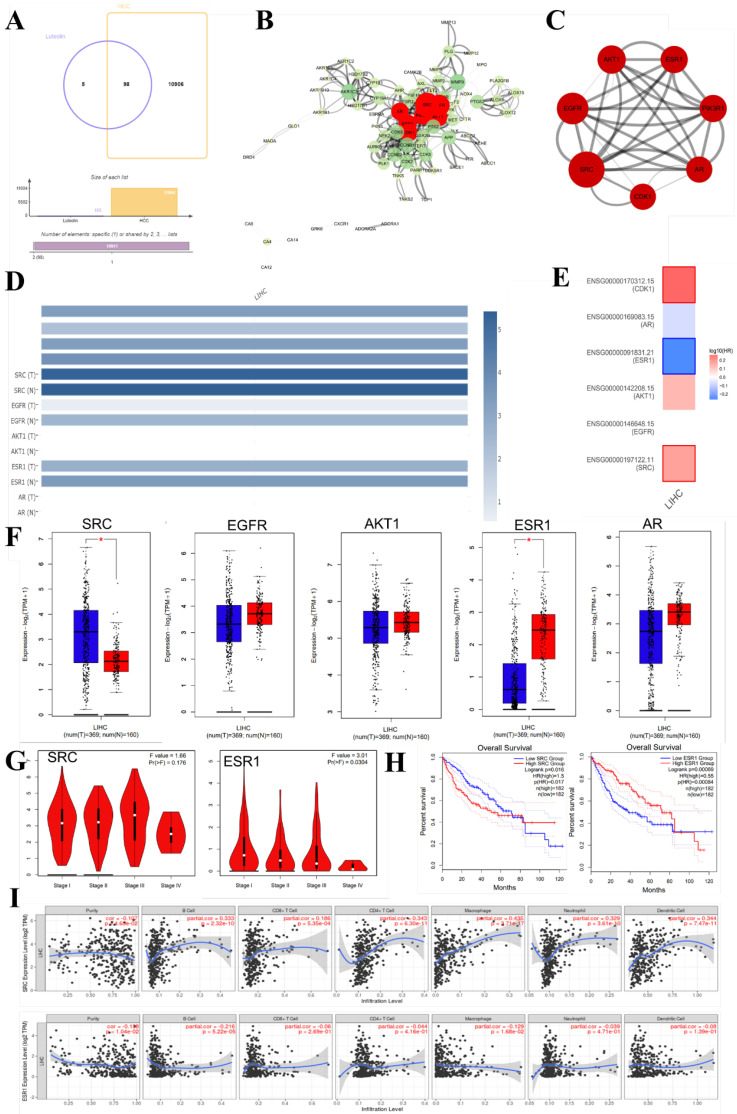

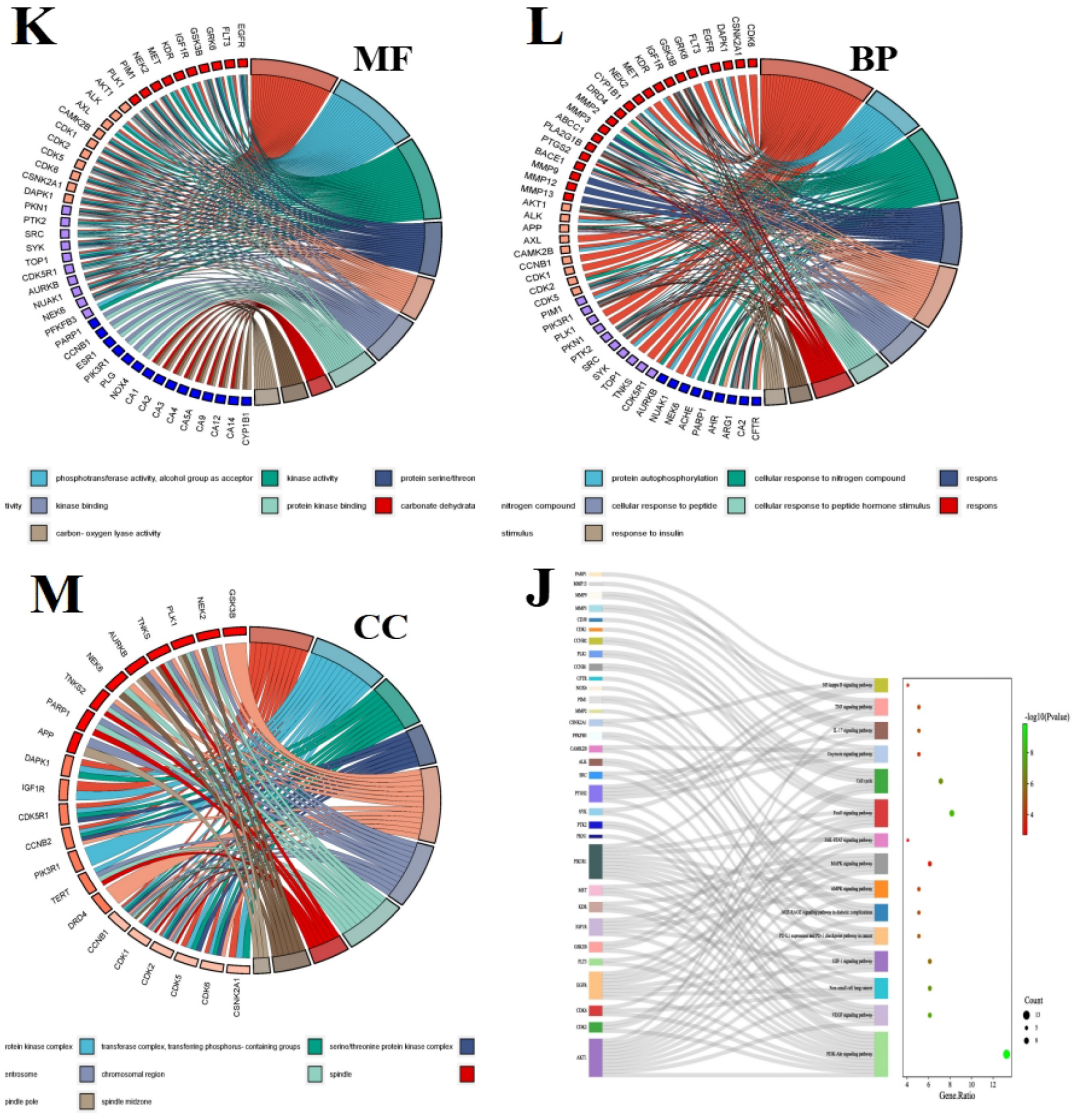
Bioinformatics analysis of potential targets of luteolin in the treatment of hepatocellular carcinoma. (A) Potential targets of luteolin in the treatment of hepatocellular carcinoma. (B) Protein interactions at potential core targets. (C) Seven core genes screened. (D) Multigene comparative analysis of candidate biomarkers based on TCGA database. (E)Survival heat map of core genes in hepatocellular carcinoma. (F) Expression of core genes based on GEPIA database. (G) Correlation analysis of differential genes in the pathological stage of hepatocellular carcinoma. (H) Influence of differential gene on survival curve of patients. (I) Correlation of differential gene SRC, ESR1 with immune cells infiltrated by hepatocellular carcinoma. (J) KEGG enrichment analysis pathway of potential core genes. (K-M) GO enrichment analysis of potential targets for luteolin in the treatment of hepatocellular carcinoma includes biological process (BP), cell composition (CC), and molecular function (MF)

In recent years, bioinformatics technology has been widely used to study the mechanism of various diseases. Network pharmacology and bioinformatics can not only systematically combine the multi-targets of drugs and diseases, but also meet the problem of few therapeutic targets and research targets in the process of clinical research through genetic research 78. Through the data mining of network pharmacology and bioinformatics analysis, we found that luteolin can interfere with the occurrence and development of hepatocellular carcinoma through multi-target, multi-gene, and multi-pathway** (Figure 6)**. After visualization, it is concluded that the main core targets of mignonette in the treatment of hepatocellular carcinoma (HCC) are SRC, EGF, AKT1, ESR1, PI3KR1, AR, CDK1, and so on. Among them, SRC and ESR1 affect the trend of the survival curve of patients with hepatocellular carcinoma and are significantly correlated with the pathological stage of patients with HCC. In addition, immune infiltration analysis showed that SRC and ESR1 were significantly correlated with six kinds of immune cells infiltrated by hepatocellular carcinoma. GO and KEGG enrichment analysis proved that the potential pathways of luteolin in the treatment of hepatocellular carcinoma include JAK-STAT, AMPK, and NF-κB.

### Signal pathway pathways of LUT treatment of HCC

When a tumor cell has a special response, the signal transmits information from outside to inside the cell. Cells respond to this kind of information. However, in the study of flavonoids, LUT plays an anti-hepatoma effect through signal transduction and protein modification. The nuclear factor-kappa B (NF-κB) signal pathway is the most important transcriptional pathway. The regulation of NF-κB can control the expression of a variety of pro-inflammatory cytokines, including cytokines, chemokines, and adhesion molecules 79. As a key effector of the Hippo pathway, YAP is activated by transport from the cytoplasm to the nucleus, which regulates gene expression and promotes tumorigenesis. Fas bind to the receptor Fas and initiate death signal transduction, which leads to apoptosis of cells expressing Fas. It has been found that luteolin can affect the survival of hepatocellular carcinoma by regulating NF- κ B, YAP, AKT/OPN, NRF2/HO-1, Fas and Fas ligand, p53, AMPK, and other signal pathways **(Table 3)**.

### The effect of LUT on HCC biological process and gene transcription

Cancer has become one of the leading causes of death because of the difficulty in treating it. Both direct and indirect risk factors can contribute to an outbreak of cancer. The emergence of gene transcription has led to significant advances in Epigenetics, with the differential expression of genes determining outcomes in cancer patients. Genome-wide screening and the discovery of high-throughput genomics have promoted the further development of proteomics, which is a breakthrough in the diagnosis and prediction of liver cancer 83. Yang Pei 56 et al. demonstrated that luteolin upregulates MIR-6809-5P expression, overexpression of miR-6809-5p inhibits HCC cell growth, and knockdown of miR-6809-5p can reverse the anti-cancer effect of luteolin. FLOT1, an essential protein for plasma membrane transport, cell division, T cell activation, and cell surface receptor signaling, also has some effects on tumor cells, and MiR-6809-5p directly targets FLOT1 in hepatoma cells 84. FLOT1 is highly expressed in a variety of tumor cells, but the down-regulation of FLOT1 by luteolin directly inhibits the growth of hepatoma cells. In most biological processes of liver cancer, multiple signaling pathways, including ERK1/2, p38, JNK, and NF-ΚB/P65, are overexpressed by miR-6809-5p or downregulated and inactivated by Flot1 85. However, in the luteolin study, extracts from Scutellaria baicalensis Georgi and Hedyotis diffusa were found to inhibit HCC cell growth and Hepatitis B virus activity *in vivo* and *in vivo* by altering circRNA-miRNA gene expression, the efficacy of these extracts may be consistent with that of Luteolin in the presence of apigenin 86. Based on network pharmacology and molecular docking techniques, Liu et al. 87 found that luteolin, as the main component of Polygonum hydropiper, was successfully combined with AKT1 to treat liver cancer, and the therapeutic effect of luteolin was verified by experiments, the levels of P-AKT mirnas are reduced in low-grade liver cancer cells and have regulatory effects on various genes such as AKT1, JUN, MAPK1, RELA, IL6, and MAPK14. In HCC cells, luteolin targets the expression of the THOC1 gene and induces DNA damage to prevent the proliferation of HCC Cells 88.

### Clinical significance and application of luteolin

With the development of natural medicine, the research on luteolin has made a great breakthrough, and the clinical significance of luteolin has been confirmed by a lot of reliable data 89. Sorafenib, a small-molecule multi-kinase inhibitor, has been approved by the US Food and Drug Administration as an oral drug for the treatment of hepatocellular carcinoma and renal cell carcinoma. But to the best of our knowledge, the combination of luteolin and sorafenib has been shown to kill cancer cells, with the study claiming that the combination increased the expression of the phosphorylated form of JNK, sP600125, a JNK inhibitor, effectively attenuated the cell death induced by combination therapy. Thus, when combined with sorafenib and luteolin to synergistically kill HCC cells through JNK-mediated apoptosis, luteolin may be an ideal drug 90. As a tyrosine kinase inhibitor, lapatinib inhibits the activation of downstream signaling pathways by blocking the activation of HER1 and HER2 tyrosine kinases, thereby inhibiting the survival and proliferation of tumors. These data suggest that the combination of lapatinib and luteolin may inhibit HER2+ human breast cancer by significantly increasing the expression of FOXO3a and NQO1 91. The results of metabolomics showed that the combination of luteolin and resveratrol could decrease the production of Glucuronic acid metabolites in patients with liver disease and increase the bioavailability of luteolin 92. In recent years, the development of key therapeutic targets of natural drugs has become an important method for the clinical treatment of tumor diseases, and the combination of luteolin, a flavonoid derivative, with clinical drugs is also of great significance for patients with hepatocellular carcinoma.

## Summary and Outlook

The effect of exploring effective compounds from natural sources for the prevention and treatment of tumors or other cancer diseases has been well proven. Flavonoids are the most widely studied anti-tumor organic compounds at this stage, and their results have provided a solid basis for clinical practice 93. In this context, luteolin, as a derivative structure of flavonoids, has received extensive attention. It promotes tumor cell apoptosis by inhibiting tumor cell growth, migration, invasion, gene expression, protein modification, etc. 94, and through the anti-tumor effects of different signal transduction processes and biological processes are becoming more and more optimistic.

Hepatocellular carcinoma is a primary malignancy tumor of the liver, and the mechanism of hepatocellular carcinogenesis is mainly concentrated in the process of hepatitis and cell regeneration 95. Liver cancer has been documented to develop in a vicious cycle of viral inflammation, alcohol-induced hepatocyte damage, or chronic liver damage caused by oxidative stress, this increases genomic instability and the risk of liver cancer 96. However, flavonoids can inhibit tumor cells through classical signaling pathways such as NF-ΚB, AKT, and AMPK, and it has been demonstrated that luteolin can achieve *in vivo* and *in vivo* anti-tumor activity by affecting levels of inflammatory factors, with no apparent toxicity to normal cells and low side effects, further validating its clinical effect 97. Non-coding RNAs, mirnas, and increase are not only involved in the initiation and development of disease but are also potential targets for drug intervention, which is consistent with studies by Yang P W et al. 86. Therefore, the next step is to search for reliable biomarkers in terms of the effect of luteolin on hepatocellular carcinoma miRNAs and lncRNAs. Based on the above data, the potential biomarkers SRC and ESR1 derived from our bioinformatics analysis may also be potential targets for luteolin in the treatment of hepatocellular carcinoma, this not only expands our hepatocellular carcinoma of luteolin treatment but also provides insight into the pathogenesis of hepatocellular carcinoma, This is consistent with the results of many previous investigators, suggesting the efficacy of already existing therapeutic targets and that new therapeutic targets may provide new strategies for the treatment of HCC. Although luteolin has shown excellent results in cancer and animal models of cancer, studies on the role of luteolin in the treatment of pharmacokinetics hepatocellular carcinoma *in vivo* need to be improved 98. Mitochondrial energy metabolism also plays an important role in the proliferation of liver cancer and other tumors, luteolin is now known to induce lethal endoparasite reticular responses, stimulus responses, and mitochondrial dysfunction in Glioblastoma multiforme cells by increasing intracellular reactive oxygen species (ROS) levels 99. As a derivative of flavonoids, luteolin has never been stopped in its research. Using nanotechnology to modify luteolin can not only improve its bioavailability but also, and the bioavailability of luteolin can be better regulated 100. In conclusion, we describe luteolin as a real source, ensuring its safety and low cost compared to synthetic cancer drugs, and describe whether luteolin can be used to prevent and treat liver diseases through different regulatory mechanisms or molecular features. As an important adjunct to the treatment of cell cancer.

## Figures and Tables

**Table 1:** The structure of flavonoids and their derivatives

Flavonoid core	Modification site	Substituent type	Derivative structure	Derivative name
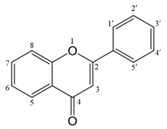	3	3=-OH	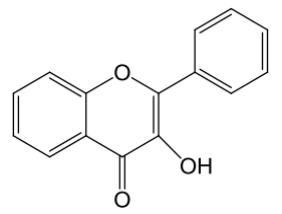	Flavonols
	3	3= 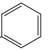	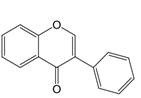	Isoflavones
	2,3	2=H 3=H	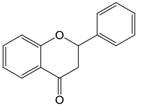	Dihydroflavonoids
	2,3	2=H 3=-OH	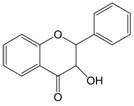	Dihydroflavonol
	2,3	2=H 3= 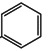	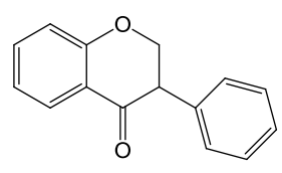	Dihydroisoflavones
	3,4	3=-OH 4=C=O→CH	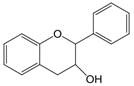	Flavan-3-ols
	3,4	3=-OH 4=C=0→C-OH	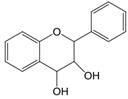	Flavan-3,4-diols
	3,4	3=-OH 4=C=0→CH=	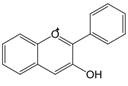	Anthocyanins
	1	1=-O-→-OH	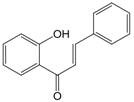	Chalcones
	1,2	1=-O-→-OH 2=CH2	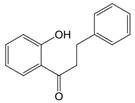	Dihydrochalcones

**Table 2:** Experimental study of luteolin compounds on tumor cells

Cancer types	Subjects cells	Mechanisms	The target gene	Have an effect	Reference
Breast cancer	MCF-7	EGFR pathway	PI3K AKT mTOR	Inhibitory signaling pathway	45,46
Colon cancer	SW620	ERK/FOXO3a pathway	ERK1/2 FOXO3a caspase-3	Cell apoptosis	47
Pancreatic cancer	PANC-1	MicroRNAs	miR-301-3p caspase-3	Gene expression	48
Lung cancer	A549	JAK/STAT1 pathway	IFN-α IFN-β JAK STAT1 MicroRNA-155	Influencing cytokines Signaling pathways Gene expression	49,50
	NSCLC	Oxidative stress	MicroRNA-34a-5p Caspase-3 Caspase-9 Bcl-2 MDM4	Cell apoptosis Signaling pathways	51
Kidney cancer	786-O	AKT pathway	JNK p38MAPK Ask1 PP2a	Inhibit cell proliferation Induce apoptosis Factor signaling	52
Gastric cancer	AGS BGC823 SGC7901	MEK pathyway	MEK ERK1/2 P21 P53 miRNA-34a	Gene expression Gene expression Signaling pathways	53
Prostate cancer	22Rv1	MicroRNAs	miR-8080 AR-V7 AR-FL Caspase-3 Caspase-7	Gene expression	54

**Table 3:** Luteolin regulates the signal pathway of HCC

Pathway	Action factor	Result	Biological process	References
NF-κB	IκBα p65 COX-2	Decreased NF-κB expression	HepG2 apoptosis	80
YAP	CXCR-4 UBTD-1	Decreased UBTD1 expression	Hep3B and Huh7 signal transduction and abnormal gene expression	81
AKT/OPN	AKT P-AKT OPN P-OPN Caspase-3	Decreased AKT/OPN expression	SK-Hep-1 apoptosis	60
NRF2/HO-1	TNF-α IL-6 NRF2 HO-1 ROS	Decreased NRF2/HO-1 expression ROS reduction	Activation of NF-κB signaling pathway in RAW264.7	80
AMPK	AMPK P-AMPK NF-κB	Decreased P-AMPK/AMP K expression	AMPK activation in HepG2 activates the NF-κB inflammatory pathway	81
p53, Fas-Fas ligand	TGF-β1 p21WAF1/CIP1 p27KIP1 Smad4	Up-regulatio of TGF-β1, p21WAF1/CIP, p27KIP1, and Smad4 gene expression	G1 arrest in Hep3B cells leads to apoptosis	82

## Abbreviations

HCC: Hepatocellular Carcinoma
LUT: Luteolin
miRNAs: Microribonucleic acids
LncRNAs: Long-stranded non-coding ribonucleic acids
ROS: Reactive oxygen species
MAPK: Mitogen-activated protein kinase
ERK: Extracellular regulated protein kinases
AKT: Protein kinase B, PKB
LDH: Lactate dehydrogenase
SOD: Superoxide dismutase
MDA: Malondialdehyde
GSH: Glutathione, r-glutamyl cysteine +glycine
FOXO3a: Forkhead box O3
NQO1: NAD(P)H:quinone oxidoreductase 1
SRC: Sarcoma
ESR1: Estrogen receptor 1
GO: Gene Ontology Consortium
KEGG: Kyoto Encyclopedia of Genes and Genomes
